# Supramolecular Structuring of Hyaluronan-Lactose-Modified Chitosan Matrix: Towards High-Performance Biopolymers with Excellent Biodegradation

**DOI:** 10.3390/biom11030389

**Published:** 2021-03-05

**Authors:** Riccardo Ladiè, Cesare Cosentino, Irene Tagliaro, Carlo Antonini, Giulio Bianchini, Sabrina Bertini

**Affiliations:** 1 Istituto di Ricerche Chimiche e Biochimiche G. Ronzoni, Carbohydrate Science Department, 20133 Milan, Italy; ladie@ronzoni.it (R.L.); cosentino@ronzoni.it (C.C.); 2 Department of Materials Science, University of Milano-Bicocca, 20125 Milan, Italy; i.tagliaro@campus.unimib.it (I.T.); carlo.antonini@unimib.it (C.A.); 3 JoinTherapeutics Srl, 35122 Padova, Italy; giulio.bianchini@jointherapeutics.com

**Keywords:** chitosan, hyaluronic acid, lactose modified chitosan, NMR, molecular weight distribution, SEM, rheology, hyaluronidase

## Abstract

Non-covalent interactions in supramolecular chemistry provide useful systems to understand biological processes, and self-assembly systems are suitable assets to build-up innovative products for biomedical applications. In this field, polyelectrolyte complexes are interesting, especially when polysaccharides are involved, due to their non-toxicity and bio-absorbability. In this work, we investigated a polyelectrolyte formed by hyaluronic acid (HA), a negatively charged linear polysaccharide, with Chitlac (Ch), a positively charged lactose-modified chitosan. The aim of the study was the investigation of a novel Ch–HA polyelectrolyte complex, to understand the interaction between the two polysaccharides and the stability towards enzymatic activity. By means of gel permeation chromatography–triple detector array (GPC–TDA), nuclear magnetic resonance (NMR), dynamic viscosity, Zeta Potential and scanning electron microscopy (SEM), the polyelectrolyte complex properties were identified and compared to individual polysaccharides. The complex showed monodisperse molecular weight distribution, high viscosity, negative charge, and could be degraded by specific enzymes, such as hyaluronidase and lysozyme. The results suggest a close interaction between the two polysaccharides in the complex, which could be considered a self-assembly system.

## 1. Introduction

Supramolecular chemistry relies on non-covalent interactions, like hydrogen bonds, hydrophobic and Van der Waals forces, and metal–ligand coordination. These interactions control many self-assembly processes, such as biological processes, and can be used to design innovative products for biomedical applications. Polysaccharides, due to their promising biomedical and biological applications, such as tissue engineering, biosensor and wound healing, are a particularly interesting class of molecules: intra- and inter-chain interactions, together with ion pairs, originate primary, secondary, tertiary, and quaternary structures, leading to supramolecular architectures [1,2]. Such architectures are suitable for a variety of applications, ranging from drug delivery to enhance the bioavailability of poorly soluble drugs [3]. Hydrophobized polysaccharides were synthetized to produce supramolecular structures in water, and their interactions with soluble proteins or other molecular assemblies, such as monolayers, black lipid membranes, liposomes and oil-in-water emulsion were studied [4].

Among the extensive number of polysaccharides available as candidates for biopolymer engineering, glycosaminoglycans (GAGs) are highly promising candidates [5]. GAGs are linear polysaccharides, which consist of repeating disaccharide units, usually include a uronic acid component (such as glucuronic acid), and a hexosamine component (such as N-acetyl-D-glucosamine).

In particular, hyaluronic acid (HA) is a linear polysaccharide with a poly-repeating disaccharide structure [(1→3)- β-D-GlcNAc-(1→4)- β-D-GlcA], which can be found ubiquitously in all vertebrate tissues extracellular matrix (ECM) [6,7]. Glucuronic acid residues contain a carboxyl group, which confers a negative charge on HA. Despite its relatively simple chemical composition, HA is involved in several biological functions, such as morphogenesis, tissue remodelling, inflammation, and tumours development and metastasis; in addition, HA contributes directly to the maintenance of tissue homeostasis and biomechanics [7]. Biocompatibility, biodegradability, high viscoelasticity, and immunoneutrality make HA an attractive polymer for biomedical and pharmaceutical applications. Frequently, HA acts as a space filler, applied to treat joint diseases such as in osteoarthritis and in eye surgery as replacement fluid, for drug delivery, and tissue engineering applications. HA is availed in viscosupplementation therapy, with the therapeutic goal to restore the viscoelasticity of synovial fluid. Indeed, HA is continuously secreted in the articular cavity and is one of the most common components of synovial liquid, giving rise to its characteristic viscoelastic properties. These rheological properties are essential for the lubrication and shock protection of healthy joints, allowing the protection of cartilage and soft tissues. In the event of traumatic and degenerative diseases, the amount of HA is lowered and synovial fluid experiences a viscosity drop, impairing joint functionality, and causing pain [8,9,10]. For applications, unmodified HA and chemically modified or cross-linked HA are used [11,12]; the derivatization of HA increases its mechanical properties and stability, which allows it to be used as a biomaterial [13,14,15].

Chitosan is also a linear polysaccharide, derived from deacetylation of chitin, the structural component of fungal cell walls, and exoskeleton of arthropods. It is composed of β-1→4 glucosamine units, with some residual interspersed N-acetyl-glucosamine residues; it is soluble in acidic solution, with a positive charge density, dependent on pH and percentage of deacetylation. Chitosan is well-known for its numerous and interesting biological properties—it is biocompatible, bioresorbable, and bioactive. Availability, safety for medical use, and biodegradability make chitosan very interesting for tissue engineering and biomaterials products. Chitosan can be further improved from the bioactive features and the physical−chemical behaviour. In particular, Chitlac (Ch) is a compound obtained by the modification of chitosan with lactitol moieties [16], in which the oligosaccharide pendant groups alter its solubility at physiological pH. The physico-chemical and biological properties of Ch are already reported in the literature [17].

Both HA and Ch are hydrolyzed by enzymes; linear HA can be easily degraded by enzymes, such as bovine testicular hyaluronidase, an endo-β-N-D-acetylhexosaminidase that hydrolyzes HA at the β (1→4)-N-acetylglucosaminide bonds [18]. Hyaluronidases simultaneously display both hydrolytic and transglycosylation activities—the optimal conditions for the hydrolysis of HA by hyaluronidase are pH 4.0 and the presence of NaCl, whereas for transglycosylation they are pH 7.0 and the absence of NaCl [19]. In the case of Ch, there is no specific enzyme; nonetheless, previous studies report that degradation can be performed by lysozyme, which is, for example, present at concentrations ranging from 4 to 13 mg/L in serum and in tears (450 to 1230 mg/L) [20].

A polyelectrolyte complex is formed when polymers with opposite charges are combined in solution. A strong complex is obtained if the anions and cations in the polymers contain strong acids and bases, or if the polyions attain their fully ionized forms and vice versa. The polyelectrolyte complexes are recently gaining attention as supramolecular carriers for controlled release of drugs and proteins [21], which is widely used in many applications such as membranes, medical prosthetics, environmental sensors, and protein separation systems [22,23]. These complexes prepared from natural polymers, such as polysaccharides, have the additional advantage of being non-toxic and bioabsorbable [24]. For example, biomaterials, constituted of a Chondroitin sulphate-Chitosan complex, have interesting biological properties, such as wound-healing acceleration and cellular assistance for skin and cartilage recovery [25]. Some applications of Chitosan and HA complexes include ophthalmic surgery, arthritis treatment, scaffolds for wound healing, tissue engineering, and the use as a component in implant materials. In particular, different authors showed that the potential of Chitosan/HA complexes coacervates in the biomaterials field [26,27,28,29,30,31]. A recent study about self-supporting multi-layered film containing a Chitosan and HA polyelectrolyte complex, showed high selectivity during the separation of water from the ethanol–water mixtures for membrane technology applications [32].

Moreover, recent studies highlighted promising results for biological and medical applications of the HA/Ch complex, such as in the treatment of osteoarthritis [33,34]; in vivo osteoarthritis treatments with a viscosupplementation containing Ch and HA showed a decrease in morphological and histolopathological cartilage damage and synovial membrane inflammation, in comparison to the treatment performed with HA alone [33]. A combination of Ch and HA-attenuated macrophage-induced inflammation, inhibited metalloproteinases expression, and exhibited anti-oxidative effects, providing interesting insights into the biological effects of mixture of these polysaccharides for the development of osteoarthritis treatments [34]. However, the literature does not provide exhaustive chemical-physical characterization of HA/Ch for a better understanding of the observed biological performances.

As such, in this work we investigated the properties of the polyelectrolyte complex to understand the interaction between HA and Ch. Specifically, the molecular weight of the supramolecular product through size-exclusion chromatography with a triple detector array (HP-SEC-TDA) was determined. The viscosity property and the molecular mobility was elucidated through dynamic viscosity and NMR diffusion ordered spectroscopy (DOSY) experiments, respectively. Finally, the stability toward enzymatic actions was evaluated.

## 2. Materials and Methods

### 2.1. Materials

Ch was provided by Join Therapeutics S.r.l. (Padova, Italy). Sodium Hyaluronate were purchased from HTL Biotechnology. Sodium azide, sodium nitrate, sodium dihydrogen phosphate monohydrate, sodium hydrogen phosphate dihydrate, trimethylsilyl-3-propionic acid (TSP), Hyaluronidase from bovine testes (400–1000 u/mg) and Lysozyme from hen egg white (93,300 u/mg) were purchased from SigmaAldrich (Milan, Italy). Deionized water (conductivity less than 0.1 μS) was prepared with an inverse osmosis system (Culligan, Milan, Italy). PolyCAL TM Pullulan std-57k (Malvern Instruments LtD, Malvern, United Kingdom). The reagent grades were ≥ 98%.

### 2.2. Molecular Weight Distribution by HP-SEC-TDA

HP-SEC-TDA was used extensively to obtain molecular weight distribution of HA, Ch, and the complex. This method does not require any chromatographic calibration and is considered to be suitable to analyze polysaccharides. Measurements for molecular weight distribution were performed on a Viscotek 305 HPLC system (Malvern Instruments LtD, Malvern, UK). The array exploits simultaneous action of refraction index detector (RI), viscometer, and Right Angle Laser Light-scattering (RALS) detector, using a method adapted from Bertini et al. [35]. To prepare the solutions for HP-SEC-TDA measurements, 10–20 mg was dissolved in a mobile phase volume, to obtain a sample concentration of ~3 mg/mL. The Ch solution was stirred for 3 h and then diluted in a mobile phase to a concentration of ~1 mg/mL. The HA solution was stirred overnight and then diluted in the mobile phase to a concentration of ~0.5 mg/mL, respectively. For the HA/Ch complex, 75 mg of Ch were dissolved in 10 mL of phosphate-buffer saline solution pH 6.9 (PBS), and stirred at room temperature to obtain a completely solubilized solution. Afterwards, 125 mg of HA were added to the solution and the complex was stirred for 12 h. Prior to the analysis of the solution, the HA/Ch complex was diluted in a mobile phase to reach 1 mg/mL sample concentration. The analyses were performed at 40 °C, using 2 x TSKPWXL columns in series (Tosoh Bioscience, 7 mm 7.8 × 30 cm). A total of 0.1 M NaNO_3_ for HA, 0.2 M NaNO_3_ for Ch, and an HA/Ch complex, both containing 0.05% NaN_3_, prefiltered using 0.22 mm filter, were used at a flow rate of 0.6 mL/min. Chromatographic profiles were elaborated using the OmniSEC software version 4.6.2. RI increments, referred to as *dn*/*dc*, were determined to enable conversion of RI values into a concentration for Ch and HA/Ch [36]. *dn*/*dc* values equal to 0.129 mL/g and 0.119 mL/g were observed for Ch and HA/Ch, respectively. For HA, the *dn*/*dc* value of 0.155 was taken from the literature [37].

### 2.3. NMR Analysis

The Ch and HA spectra were obtained with a Bruker AVANCE IIIHD 500 MHz spectrometer (Bruker, Karlsruhe, Germany) equipped with a 5 mm BBO probe, at 343 K. Spectra were processed with BrukerTopspin software version 4.0.6.

About 30 mg of sample were dissolved in 3 mL of deuterium oxide (D2O) with 0.002 %TSP and subsequently 0.6 mL were transferred into a 5 mm NMR tube. The HA sample was stirred overnight to ensure a complete solubilization before transfer in an NMR tube (Bruker, Karlsruhe, Germany) for the analysis. ^1^H NMR spectra were acquired with pre-saturation of residual HDO, using 64 scans, 12 s relaxation delay, and a number of time-domain points equal to 32k. For the Ch sample, the ^1^H-^13^C HSQC spectra were acquired using 16 scans, 5s relaxation delay, ^1^J_C-H_ 150 Hz.

The HA/Ch complex spectra were acquired with a Bruker AVANCE NEO 500MHz spectrometer (Bruker, Karlsruhe, Germany) equipped with a 5 mm TCI cryogenic probe at 303K for ^1^H NMR and Diffusion Order NMR Spectroscopy (DOSY). Spectra were processed with Bruker Topspin software version 4.0.6. Ch and HA were dissolved separately in a PBS deuterium volume (mL), which allowed us to reach 7.5 mg/mL and 12.5 mg/mL, respectively. Then, 500 µL of both solutions were taken and mixed, it was vortexed to obtain a homogeneous solution.

Spectra were acquired with pre-saturation of residual HOD using 64 scans, 5 s relaxation delay, and a number of time-domain points equal to 65 k.

DOSY experiments were acquired using the 2D-stimulated echo sequence with bipolar gradient pulse for diffusion. The gradient pulse (δ) and the diffusion time (∆) were set to 5 ms and 300 ms, respectively. The 2D DOSY experiments were run with gradients varied linearly from 5 to 95% in 32 steps, with 16 scans per step. The diffusion coefficients D were extracted using the Bruker Dynamics Center 2.5 (Bruker, Karlsruhe, Germany).

### 2.4. Zeta Potential Analysis

Zeta Potential (Zp) of HA, Ch, and the HA/Ch complex were measured using the Zetasizer Nano ZS (Malvern, Worcestershire, United Kingdom), with a fixed scattering angle of 173° and a 633-nm helium–neon laser. Data were analyzed using the Zetasizer software version 7.11 (Malvern Instruments LtD, Malvern, UK). For the analysis, Ch and HA solutions were diluted in PBS to reach the concentration of 7.5 and 12.5 mg/mL, respectively. In the case of the HA/Ch complex, 75 mg of Ch were dissolved in 10 mL of PBS and stirred at room temperature, to obtain a completely solubilized solution. Then, about 125 mg of HA were added to the solution and the complex was stirred for 12 h. Disposable plastic cuvettes DTS1070 were used for the Zp analysis.

### 2.5. Rheological Properties

The rheological properties of HA, Ch, and the complex were studied using a Modular Compact Rheometer MCR 92 (Anton Paar GmbH, Graz, Austria), equipped with a 50-mm-diameter cone–plate geometry, with a cone angle of 1°. For all tests, the temperature and the gap between the plates were kept constant 20 °C and 0.98 mm, respectively.

Viscosity measurements were performed in rotation mode, they were investigated in the range of 0.1–100 s^−1^ and ten points per decade were acquired.

The sample viscoelastic behaviour was investigated in the oscillation mode, to determine the storage modulus G’(ω) and the loss modulus G”(ω). First, preliminary tests were conducted to determine the upper amplitude limit of the linear viscoelastic region (LVE), testing the samples over an extended strain field (0.01–100%). Second, after the LVE was determined (2% for HA and HA/Ch and 20% for Ch), the samples were tested by performing a frequency sweep test over the 0.628 rad/s–628 rad/s (i.e., 0.1–100 Hz) frequencies, at a constant strain. Data were elaborated with RheoCompass™ software.

For the analysis, the same solutions as for Zp were tested. Before the analysis, solutions were sonicated to reduce air bubbles.

### 2.6. SEM Analysis

Scanning Electron Microscopy (SEM) analysis was performed with Zeiss Gemini 500 Field-Emission SEM (Carl Zeiss Microscopy, Oberkochen, Germany) at 5 kV. Polysaccharide samples of 50 mg were freeze-dried in a 100 mL Falcon tube. The dried samples where broken with tweezers, deposited on SEM adhesive tape and sputtered with gold to enhance conductivity.

### 2.7. Enzymatic Degradation Procedure

CH (solution A): 75 mg of sample were dissolved in 10 mL of PBS and stirred to obtain a completely solubilized solution. HA (solution B): 62 mg of HA sample were dissolved in 5 mL of PBS and stirred for 16 h. HA/Ch complex (solution C): 62 mg HA were dissolved in 5 mL of solution A. The final concentration of the polysaccharide (HA/Ch) was about 20 mg/mL (7.5 mg/mL CH + 12.5 mg/mL HA). For enzyme solution preparations, about 15 mg of hyaluronidase were dissolved in about 1.5 mL of deionized water and stirred for 1 h (10 mg/mL). Lysozyme: 75 mg of lysozyme was dissolved in about 1 mL of deionized H_2_O and stirred for 1 h (75 mg/mL). The solutions were stirred at 38 °C in an oil bath for about 2 h, before adding the enzyme solution. Different aliquots of solutions were collected at different times (15 min, 30 min, 1 h, 2 h, 4 h, 6 h, 24 h) and heated at 100 °C for 5 min, using a thermo-shaker, (BioSan, Riga, Latvia) to denature the enzyme. The solutions were diluted to different concentrations for HP-SEC-TDA analysis. The Ch solutions were diluted to 2 mg/mL with the mobile phase, whereas the solutions containing HA or HA/Ch were diluted to 0.5 mg/mL (calculated on the total amount of polysaccharide in solution) with the mobile phase. Before the HP-SEC-TDA analysis, all solutions were filtrated to remove the precipitated enzyme (LLG-Syringe filter, CA pore size 0.20 µm, Ø 13 mm).

## 3. Results

### 3.1. Molecular Weight Distribution

**Figure 1:**
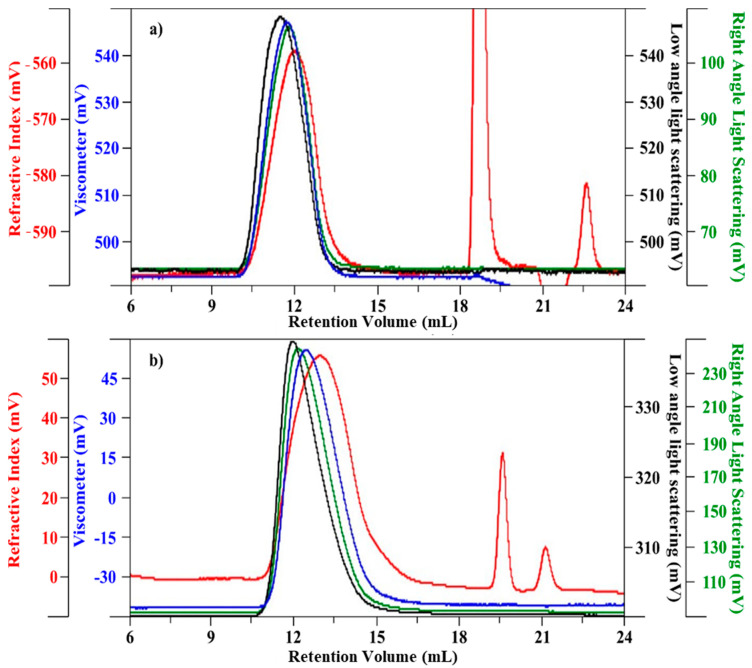
Chromatographic profile (red—refractive index; black—low laser light scattering; green—right angle light scattering; blue—viscometer). (**a**) HA, and (**b**) Ch.

Chromatographic profiles of HA and Ch are shown in Figure 1a,b, respectively. The samples had an elution time between 10 and 15 mL, with a large a-symmetric bell-shape chromatographic peak, caused by a high polydisperse index.

Values of weight-average molecular weight (Mw), number-average molecular weight (Mn), and molecular-weight dispersity (Ð; Mw/Mn) are reported in Table 1. In addition, values of the intrinsic viscosity value (η), hydrodynamic radius (Rh), and Recovery %, derived from TDA, are also reported. To estimate the Recovery %, the OmniSEC software computes actual concentration in each chromatographic slice, based on the *dn*/*dc* value, and a detector constant for the RI. The values of *a* and *log k*, corresponding to the slope and intercept constants of the Mark-Houwink curve, respectively, are also reported. The results refer to the mean values of duplicate injections.

For the HA samples, a recovery value of 90% indicated that no material remained adsorbed in the columns, considering that the water content in the sample was about 10% *w*/*w*; for Ch, the values were close to 100%, due to the low water content in the sample. The Ð value for HA was lower than that for Ch, indicating low molecular weight dispersion. However, the values were relatively high, indicating the presence of chains of different length.

**Figure 2:**
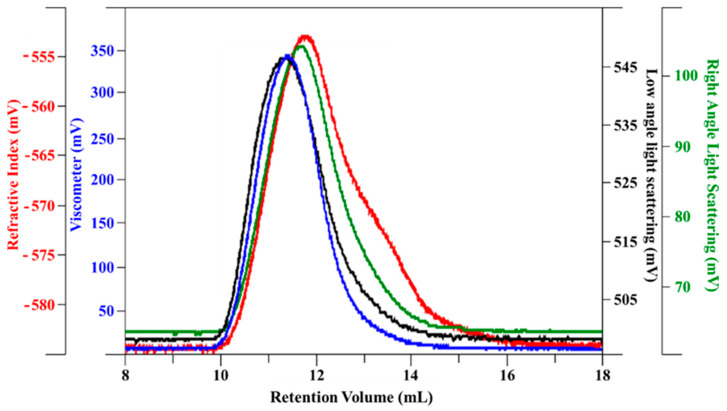
Chromatographic profile (red—refractive index; black—low laser light scattering; green—right angle light scattering; blue—viscometer) of the HA/Ch complex.

The HA/Ch complex (0.75:1.25 *w*/*w*) molecular weight was also determined by the same technique—results are visualized in Figure 2 As expected, due to very similar molecular weight, the two polysaccharides were eluted together, so it is important to evaluate the *dn*/*dc* of the complex. From analysis on several dilutions of a HA/Ch complex, the refractive index increment (*dn*/*dc*) was determined as 0.118, through a linear regression.

Using 0.118 as *dn*/*dc*, the molecular weight analysis results are reported in Table 2. Molecular weight, dispersity, and hydrodynamic radius of the complex (Table 2) remained quite similar to those of the original polymers (Table 1). Generally, through gel permeation chromatography analysis, the average molecular weight of polymers increased due to cross-linked reactions or derivatizations. In the HA/Ch complex, only weak interactions between the two polysaccharides (hydrogen bonds and Van der Waals forces) are present, which are influenced by high salt concentration of mobile phase.

### 3.2. NMR Characterization

Most of the properties of chitosan and its derivates, such as solubility and biodegradability, depend on the proportion between acetylated and non-acetylated glucosamine units, corresponding to degree of acetylation (DA), and eventually substitution degree (DS). NMR spectroscopy is one of the most accurate methods for determining the degree of acetylation for chitosan [38] and DS for Ch compounds [39].

**Figure 3:**
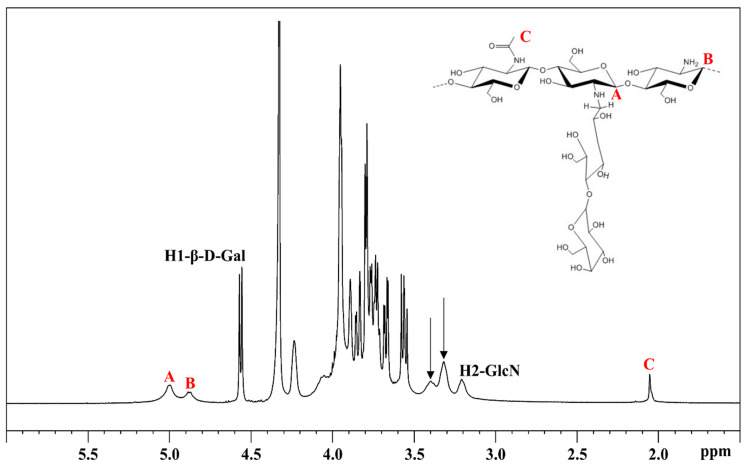
^1^H NMR spectrum of Ch in D_2_O at 343 K.

Figure 3 shows the ^1^H NMR spectrum of a Ch sample. The characteristic peaks are located at 3.32 and 3.40 ppm (black arrows Figure 3)—such signals represent the H2 of substituted deacetylate units (H2-NHR) and the hydrogens of the CH_2_ group involved in the amide bond (-NH-CH_2_-), respectively. The H2 of unsubstituted deacetylate units (H2-GlcN) appears at about 3.21 ppm. The peaks in the anomeric region, A (5.00 ppm) and B (4.88 ppm), in Figure 3 are attributed to H1 of the substituted and unsubstituted deacetylate units, respectively. These peaks and the CH_3_ signal of the acetyl group at 2.06 ppm (C, Figure 3) were used for the DA and DS calculation. In the anomeric region, the two sharp peaks were attributed to the anomeric protons of β-galactose side chains, at 4.57 ppm [17].

By assigning 300 to the integral of the signal related to the methyl group of acetyl (about 2.06 ppm), the percent N-acetylation (%*DA*) and substitution degree (%*DS*) were evaluated, using Equations (1) and (2).

(1) $$\%DA\;=\;\frac{\frac{C}{3}}{A+B+\frac{C}{3}}\;\cdot 100$$

(2) $$\%\;DS\;=\;\frac{A}{A+B}\;\cdot \left(100-\%DA\right)$$

From the integral values it was seen that in the Ch sample, *DA* and *DS* were 7.5% and 59%, respectively.

**Figure 4:**
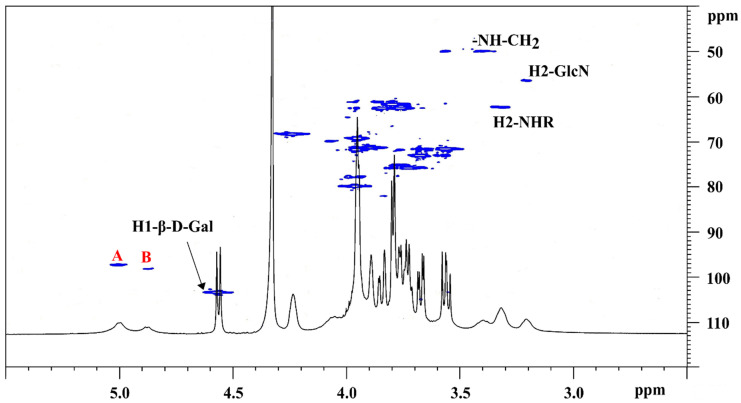
^1^H-^13^C HSQC NMR spectrum of CH in D_2_O at 343 K.

To confirm the peak attributions and to verify that the integrated signals involved in quantification corresponding to the unique function units, the ^1^H-^13^C HSQC spectrum was acquired (Figure 4). The 2D spectrum confirmed that the anomeric and methyl peaks in the ^1^H spectrum corresponded to one peak in the ^13^C dimension, and that ^13^C chemical shifts were consistent with the attributed signals of product [17].

Pulsed field gradient (PFG) diffusion ordered spectroscopy (DOSY) is the translational diffusion of dissolved molecules. In addition to the overall molecular size and shape, the diffusion coefficient magnitude provides direct information on molecular dynamics, including intermolecular interactions [40], aggregation, conformational changes [41,42], and viscosity [43]. DOSY processing is a particularly suitable technique for complex samples, since it provides a direct correlation of translational diffusion to the chemical shift in the second dimension. Therefore, a prior separation of complex components is not required [44,45]. A DOSY experiment is represented in a 2D spectrum, with chemical shift along one axis and the diffusion coefficients along the other [46,47].

**Figure 5:**
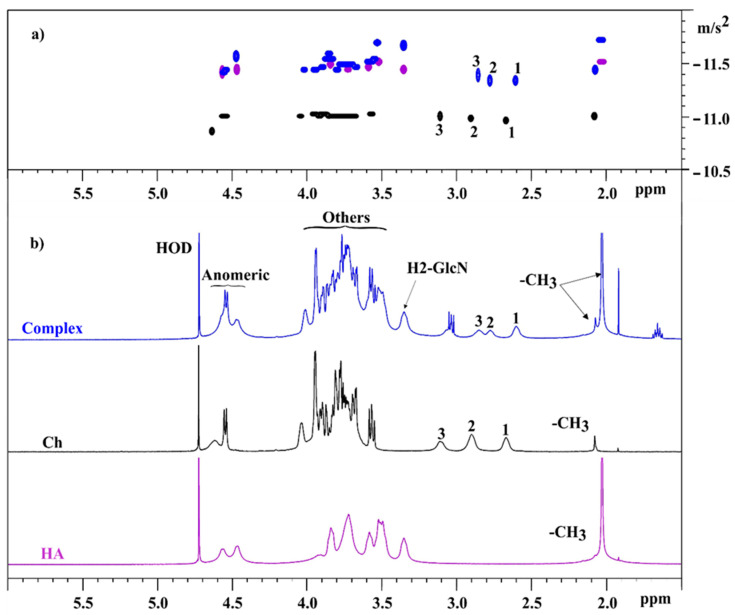
(**a**) DOSY and (**b**) ^1^H spectra of HA (purple), Ch (black), and complex (blue).

In this study, DOSY was used to evaluate the interaction between HA and Ch in the complex. The outcomes of the DOSY analysis for all samples are visualized in a 2D map in Figure 5a.

For the HA and Ch samples, there is only one population with the same diffusion coefficient, due to a unique molecular weight. For the HA/Ch complex, the results showed different diffusive fronts, due to different hydrodynamic radius of (5a) the HA resonances, and (5b) the resonances due to Ch moiety. The different chemical shift for 1, 2, and 3 signals were related to different small pH of solutions in the presence of HA. Usually, chemical shift of main chain protons, involved in the amino bond (H2) are highly sensitive to the protonation equilibrium [17]. The D values, shown in Table 3, were determined for the peaks corresponding to the components of the complex.

The average D value of HA was lower than Ch, despite having similar Mw values. In this case, DOSY was particularly successful in distinguishing among different molar diffusivities, due to the different hydrodynamic radii, confirming the HP-SEC-TDA results. In the HA/Ch complex, the D values of the HA component remained almost constant, meanwhile the Ch diffusion coefficients increased, with values analogous to HA.

### 3.3. Rheological Properties

Rheology as reported by Ambrosio et al. [48], is a useful tool to explore the relationships between the mechanical behavior and chemical properties of HA, or other biopolymer solutions. In our study, the flow behavior and viscoelastic measurement were evaluated to understand the difference in the rheological properties of HA and Ch solutions with their complex.

**Figure 6:**
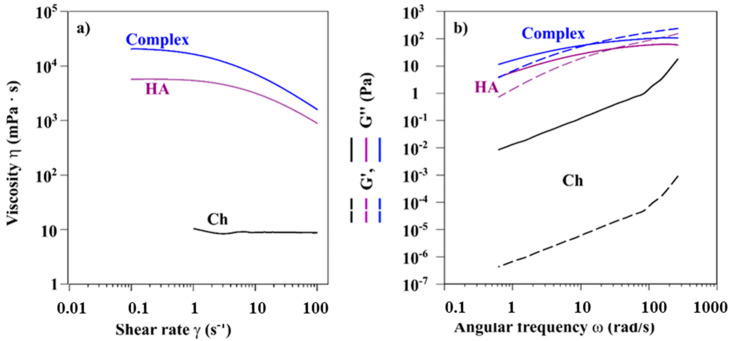
(**a**) Viscosity curves of the Ch, HA, and HA/Ch complex. Due to low viscosity, data for Ch were only acquired in the range (1–100) s^−1^ (**b**). Frequency sweep test of the Ch (γ = 20%), HA, and the HA/Ch complex (γ = 2%).

The variation of the viscosity as a function of the shear rate were acquired; in Figure 6a the viscosity curves, with a shear rate (γ) ranging from 0.1 s^−1^ to 100 s^−1^, are reported.

HA and Ch (black and purple curves in Figure 6a, respectively) showed different behaviours. Ch viscosity remained constant at a value of 8.8 mPa s over the entire tested shear rate range, thus showing ideal viscous flow behaviour. Such behaviour was mainly related to the sample concentration and substitution degree of chitosan—according to previous results [49]. The pseudoplasticity was inversely proportional to DS and directly correlated to the concentration of sample. For HA, viscosity was shear-dependent, viscosity decreased as γ increased, in agreement with a pseudoplastic behaviour [48]. For the complex, shear-thinning behaviour was also observed. Note that the viscosity values were higher than that for pure HA.

The rheological tests were also performed in the oscillation mode, to evaluate both the storage modulus (G’) and the loss modulus (G”). The strain sweep test (data not shown) was initially performed to evaluate the LVE zone, where the intrinsic sample structural properties are independent of the applied strain:2% (HA and Complex) and 20% (Ch) strain value was selected for subsequent frequency sweeps tests. In Figure 6b, values of G’ and G’’ as function of the angular frequency for the Ch, HA, and the complex are reported. In the investigated frequency range (from 0.628 rad/s to 628 rad/s), the mechanical spectrum of Ch showed that G” was greater than G’, with both moduli strongly dependent on the frequency, as typically a liquid-like (viscous) behavior dominates over the solid-like (elastic) character. Differently, HA and HA/Ch presented a “weak gel” or viscoelastic behavior with G’ and G”, which became less dependent on the frequency and the crossover point, when G’ = G”, was observed. This suggests that at high frequencies, when G’ > G”, the material show a predominant solid-like behavior.

The angular frequency values, ω**_c_**, and the corresponding G’ = G” values to the crossover point data are reported in Table 4.

### 3.4. Zeta Potential

Zp, a parameter typically obtained by model-dependent transformation of the measured electrophoretic mobility, is a parameter used to estimate the magnitude of the electrostatic repulsion or attraction between particles. By measuring the surface charge, the stability of nanosuspensions, particles, and polymers in solution can be determined [50]. A large positive or negative Zp value, with values >+25 mV or <−25 mV, indicate good stability due to electrostatic repulsion of individual particles; a lowZp value can result in particle aggregation and flocculation due to the van der Waals attractive forces [51,52,53]. The Zp is frequently used to understand polysaccharide–protein complexation [54].

In this study, the Zp values of solutions of the Ch, HA, and HA/Ch complex were determined; the results are reported in Table 5. The Ch and HA samples were studied at concentrations of 7.5 and 12.5 mg/mL, respectively, which reflect the concentrations of the individual samples in the complex.

HA showed a negative Zp value, which was consistent with its anionic nature to the presence of carboxylic groups. Low zeta potential values provided an electrostatic repulsion that led to the formation of suitable stability in solution. Else, the Zp value of Ch was zero, indicating a slightly positive surface charge, close to zero, in agreement with the chemical structure. When the two polysaccharides were mixed, the Zp of the complex was negative and homogeneous. The value of about −23 mV meant that the complex in solution was stable without any macro-aggregation phenomena that could induce precipitation.

### 3.5. SEM

SEM analysis was performed to assess the morphological features of the HA/Ch complex [55], after a freeze-drying process. In the first step of freeze-drying, the water-based solutions (or suspensions) were first frozen—ice nucleated and concentrated the solute (or suspended matter) in the regions between the growing crystals. In the second step, ice was removed via direct sublimation at low pressure, avoiding the intermediate liquid phase, and leaving a porous morphology. The process is widely used to create porous materials using biopolymer [56,57,58]. The final morphology depends on the specificmaterial, and is controlled by many parameters of the freezing process [59], including sample size and shape, cooling rate, cooling temperature, process time, etc.

**Figure 7:**
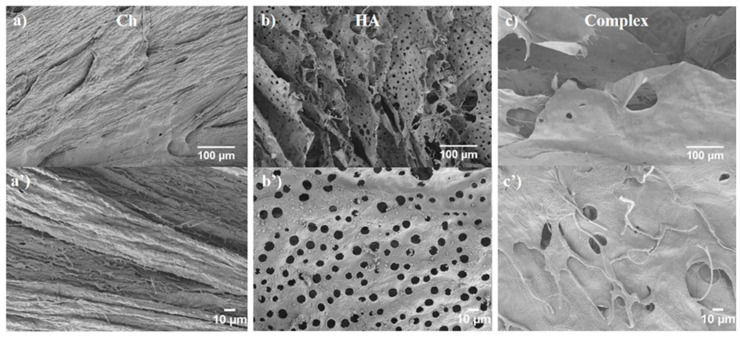
SEM analysis of (**a**) Ch (magnification 150×); (**a’**) Ch (magnification 500×); (**b**) HA (magnification 150×); (**b’**) HA (magnification 500×); (**c**) Complex (magnification 150×); and (**c’**) Complex (magnification 500×).

Figure 7 illustrates the specific characteristics of the HA/Ch complex that were compared to the single components of Ch and HA.

Ch (Figure 7a,a’) showed a compact structure with a fibrous appearance, causing a rough texture on the Ch surface. HA presents even more clearly a lamellar structure (Figure 7b,b’), which is characteristic of an ice-templated process—the structure was oriented parallel to the temperature gradient (i.e., from the skin to the sample centre), along which the ice crystal grew. HA walls were characterized by a pore regular pattern, with a characteristic diameter of ~10 µm. Such pores might also be related to ice-templating, with holes forming due to the secondary crystal growth, perpendicular to the primary crystal growth direction.

The HA/Ch composite (Figure 7c,c’) showed a compact and smooth surface, where fibers were visible on the top of the surface. The material was made of a lamellar structure with the holes between 10 and 20 µm as diameter. Similar to Zhang et al. [60], who investigated the structure of porous chitosan/HA/sodium glycerophosphate hydrogel systems, the material appeared homogeneous overall, and phase separation at the micro-scale was not observed. As such, we could confirm good compatibility between Ch and HA in the complex.

### 3.6. Enzymatic Degradation

**Figure 8:**
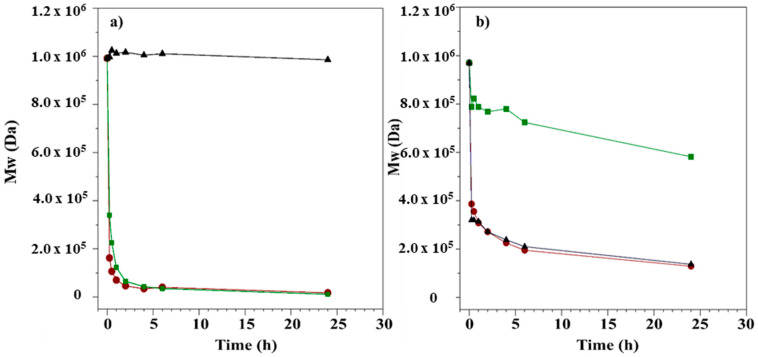
(**a**) HA and (**b**) Ch kinetic degradation with HAse (green curves), Lys (black curves), and a mix of enzymes (red curves) over 24 h.

The HA/Ch enzymatic degradation with lysozyme and hyaluronidase was studied. Such tests provide information on the polysaccharide stability in a physiological environment. The samples incubated at different times were analyzed. The Mw values vs. time, determined by chromatographic elaboration, are shown in Figure 8.

The HA treatment (Figure 8a, green line) resulted in a substantial reduction of Mw indicating depolymerization, and such depolymerization took place very quickly during the initial 3 h, this indicated that the enzymatic hydrolysis was an endo-action. After 3 h, degradation slowed down, probably due to the inhibition of the enzyme activity by the end products, and the reaction was completed after 24 h, at the end of which a reduction of the Mw of 98% was observed (from 992 kDa to 14 kDa). In the case of Lys (Figure 8a, black line), the enzyme did not have any degradation effect, and Mw remained constant for the incubation time (24 h). As expected, the presence of Lys in complex with HAse (Figure 8a, red line) had a mild effect on the HA hydrolysis, showing a slightly different initial degradation rate, but the end point was the same.

For Ch, in the enzymatic degradation with Lys (Figure 8b, black line), the Mw decreased slowly and, after 24 h of incubation, the value was reduced by 86% (from 970 kDa to 137 kDa); with HAse (Figure 8b, green line) a small reduction of Mw was observed, at about 20%, which was in agreement with the lack of a specific substrate for this enzyme. The decrease of Mw was the same with the complex of enzymes than with Lys alone—as expected, the “driving force” for the degradation of Ch was Lys but the presence of hyaluronidase seemed to induce an effect on the first part of hydrolysis process.

To study how the interactions between HA and Ch could affect the enzymatic activity, the complex was hydrolyzed. The results with HAse and Lys individually and finally with enzymatic complex are shown in Table 6. All chromatograms of solutions until 6 h of incubation present only one peak, derived from HA and Ch, due to the very similar molecular weight. Consequently, the Mw and [η] values are related to the complex, imposing the *dn*/*dc* of 0.118 (see HP-SEC-TDA results section).

After 6 h, the Mw was reduced by about 70% with HAse, and 20% and 92% with Lys and with HAse + Lys, respectively. Therefore, the enzyme activity was not affected by the combination of the two polysaccharides.

**Figure 9:**
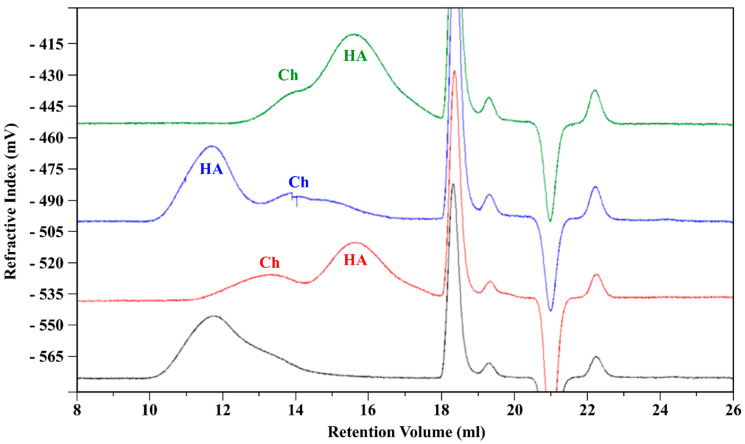
RI signal from the HP-SEC-TDA analysis of the HA/Ch complex at t = 0 (black) and after degradation t = 24 h with HAse (red), Lys (blue), and a mix of enzymes (green).

After 24 h of incubation, the chromatograms present two distinct peaks (Figure 9), one related to the HA component and the other one attributed to Ch, so Mw and [η] for each polysaccharide could be determined, using the specific *dn*/*dc* values. The results are presented in Table 7.

The Mw values were very similar to the results obtained for the solution aliquots containing only HA or Ch, at 24 h, suggesting that the co-presence of both polysaccharides did not affect the enzymatic action.

## 4. Discussion

The polyelectrolyte complex in the aqueous medium interacts and self-assembles by electrostatic interactions. Therefore, the development of new stable formulations, constituting polyelectrolyte, requires an in-depth characterisation of the interactions to obtain biodegradable and biocompatible products with unique properties, without losing the polymers inherent characteristics. In the present work, efficacies analytical approaches were set up to characterize the HA/Ch complex and to evaluate its stability toward enzymes.

The interaction between HA and Ch was affected by the structural peculiarity of each polysaccharide. With regards to HA, the chemical structure was well-defined, meanwhile for Ch, the DS and DA were variable. Generally, the proportion of glucosamine residues in chitosan had a significant influence on their various properties. In particular, the distribution of N-acetyl groups along the biopolymer chain might influence the solubility of the polymer and the inter-chain interactions, due to the hydrogen bonds and the hydrophobic character of the acetyl group. Moreover, the derivatization of chitosan with alkyl groups induced high mobility side chains, causing them to be highly hydrated. It follows that the DS and DA of Ch are important parameters for the property of the polysaccharide in solution and for the interaction with HA. A schematic representation of how Ch and HA interact to form a complex is given in Figure S1.

A quantitative ^1^H NMR method was applied to determine the DA and DS of Ch. The Ch sample with DA and the DS values of 7.5% and 59%, respectively, is soluble in water at neutral pH and it presents a low viscosity, as compared to the same molecular weight of HA, as shown by the HP-SEC-TDA results (Table 1) and the viscosity curves (Figure 6a). HA was approximately 6 times more viscous in terms of intrinsic viscosity than Ch, and the hydrodynamic radius for the negative charge polysaccharide was double, as compared to Ch, although the molecular weights were quite similar. This behavior was confirmed by the DOSY NMR experiment (Table 3). Indeed, the average D values of HA were lower than those of Ch, indicating a lower mobility due to higher viscosity. When the two polysaccharides were mixed, anaverage molecular weight distribution between HA and Ch was obtained, which maintained a high intrinsic viscosity value (Figure 2, Table 2).

The homogeneity of the complex was demonstrated by the Zp measurements (Table 5)—one Zp value was observed and through the comparison of the results, the HA/Ch complex Zp was about –23 mV, lower than that obtained for the HA alone (−34 mV), indicating that the negative charges of HA were partially reduced in the presence of Ch, due to the phenomena of secondary interaction between the polysaccharides. The strict interaction between HA and Ch was also demonstrated by DOSY:the D values of HA and Ch in the complex were similar (Table 3).

It clearly appears that by mixing the two polysaccharides, an interesting modulation of the viscoelastic properties could be easily obtained (Figure 6b). The mechanical spectra of the solutions prepared using only HA or Ch represent two different behaviors—the HA solution had a relevant viscoelastic character, whereas the Ch sample presented a more evident liquid/elastic character. Finally, the HA/Ch solution showed the highest values of both elastic and viscous moduli, with a shift toward higher frequency of the crossover point (Table 4).

SEM analysis on the Ch/HA composite supported the data that suggest an intimate mixing of the components, revealing characteristic features of the individual polysaccharide, such as lamellae, fibers, and pores.

We demonstrated that HA and Ch, solubilized together, can generate secondary interactions that modified the chemico-physical properties of polysaccharides. To verify if these phenomena could affect the biocompatibility, the stability toward enzymatic actions was evaluated, comparing the results obtained from a single component preparation and the complex of HA/Ch. While the HA was fully degraded by Hyaluronidase and was not hydrolyzed by Lysozyme, Ch was mildly degraded by Hyaluronidase, and was fully hydrolyzed by Lysozyme. Using the Hyaluronidase and Lysozyme complex with each polysaccharide, the Mw values were the same for those obtained with the specific enzymes. This meant that there was no cooperative effect in the degradation process. Analyzing the HA/Ch complex, HA hydrolysis was not modified by Ch, wheras Ch appeared more resistant to enzyme hydrolysis in presence of HA. Our hypothesis suggests that the penetration of the enzymes to the network HA/Ch, and in particular, the availability of Ch molecules to the active site of the enzymes were slightly hindered; however, but the hydrolysis processes was maintained.

## 5. Conclusions

In this study, we investigated the supramolecular structuring of a HA/Ch complex using diverse analytical methods. The HA/Ch solution was characterized in terms of physicochemical properties. The supramolecular complex showed interesting rheological properties and promising preliminary evidence for biocompatibility—monodisperse molecular weight distribution, high viscosity, negative charge, degradation by specific enzyme, such as Hyaluronidase and Lysozyme. Due to the wide range of applications in biomedicine and biotechnology, the development of such polyelectrolyte complexes is of scientific and technological interest. Such an analytical approach would facilitate the development of new formulations, demonstrating the interactions with the different components.

## Figures and Tables

**Table 1:** Main results of the HP-SEC-TDA analysis for the HA and Ch samples.

Sample	Mw (kDa)	Mn (kDa)	Ð	a	log K	[η] (dL/g)	Rh (nm)	Recovery%
HA	992	660	1.5	0.69	−2.9	17.4	63	90
Ch	1020	554	1.8	0.68	−3.6	3.0	34	96

**Table 2:** HP-SEC-TDA HA/Ch complex results.

Sample	Mw (kDa)	Mn (kDa)	Ð	a	log K	[η] (dL/g)	Rh (nm)
HA/Ch complex	1099	619	1.8	1.28	−6.8	7.9	49

**Table 3:** DOSY results of HA, Ch, and HA/Ch complex (D = H_2_O 2.25 × 10^−9^ m^2^/s; D = TSP: 6.50 × 10^−10^ m^2^/s).

	HA		Ch		HA/Ch Complex			
Peak	^1^H ppm	D (m^2^/s)	^1^H ppm	D (m^2^/s)	^1^H ppm		D (m^2^/s)	
CH_3_	2.03	1.48 × 10^−12^	2.07	6.89 × 10^−12^	2.03	2.07	6.35 × 10^−13^	1.86 × 10^−12^
1	-	-	2.73	7.05 × 10^−12^	2.60		2.64 × 10^−12^	
2	-	-	2.96	7.11 × 10^−12^	2.77		2.89 × 10^−12^	
3	-	-	3.14	7.53 × 10^−12^	2.85		3.05 × 10^−12^	
H2-GlcA	3.36	1.61 × 10^−12^	-	-	3.35		7.51 × 10^−13^	
Anomeric	4.40–4.60	1.72 × 10^−12^	4.50–4.55	6.60 × 10^−12^	4.40–4.60		1.86 × 10^−12^	
others	3.51–3.84	1.51 × 10^−12^	3.54–4.04	6.65 × 10^−12^	3.54–4.04		1.60 × 10^−12^	

**Table 4:** Rheological measurements—evaluation of G’ and G” crossover.

Sample	Angular Frequency ω_c_ (rad/s)	Crossover Point G’ = G” (Pa)
Ch	n.d. ^1^	n.d. ^1^
HA	30	44
HA/Ch	13	63

^1^ value not detected in the frequency range investigated.

**Table 5:** Zp values for HA, Ch, and complex.

Sample	Concentration (mg/mL)	Zp (mV)
HA	12.5	−33.74
Ch	7.5	0.03
HA/Ch complex	20.0	−23.28

**Table 6:** HP-SEC-TDA results of the HA/Ch complex degradation.

	HAse		Lys		HAse + Lys	
Time (h)	Mw (kDa)	[η] dL/g	Mw (kDa)	[η] dL/g	Mw (kDa)	[η] dL/g
0	1099	10.0	1099	10.0	1099	10.0
0.25	406	2.0	902	7.3	192	1.6
0.30	377	1.7	875	7.3	167	1.2
1	370	1.5	884	7.4	150	1.0
2	352	1.4	904	7.6	128	0.9
4	345	1.3	878	7.4	104	0.7
6	331	1.3	884	7.7	95	0.6

**Table 7:** HP-SEC-TDA results of the HA/Ch complex degradation after 24 h.

	HAse		Lys		HAse + Lys	
	Mw (kDa)	[h] dL/g	Mw (kDa)	[h] dL/g	Mw (kDa)	[η] dL/g
HA	16	0.4	955	15	19	0.3
Ch	620	2.3	155	1.6	220	1.1

## Data Availability

The data that support the findings of this study are available from the corresponding author upon reasonable request.

## Supplementary Materials

The following are available online at https://www.mdpi.com/2218-273X/11/3/389/s1, Figure S1: Schematic interaction between HA and Ch in Phosphate buffer saline.
