# Supplementary figures and images for: Supramolecular Structuring of Hyaluronan-Lactose-Modified Chitosan Matrix: Towards High-Performance Biopolymers with Excellent Biodegradation
Source: Biomolecules. 2021 Mar 5;11(3):389. doi: 10.3390/biom11030389 (PMC8000860; doi:10.3390/biom11030389)

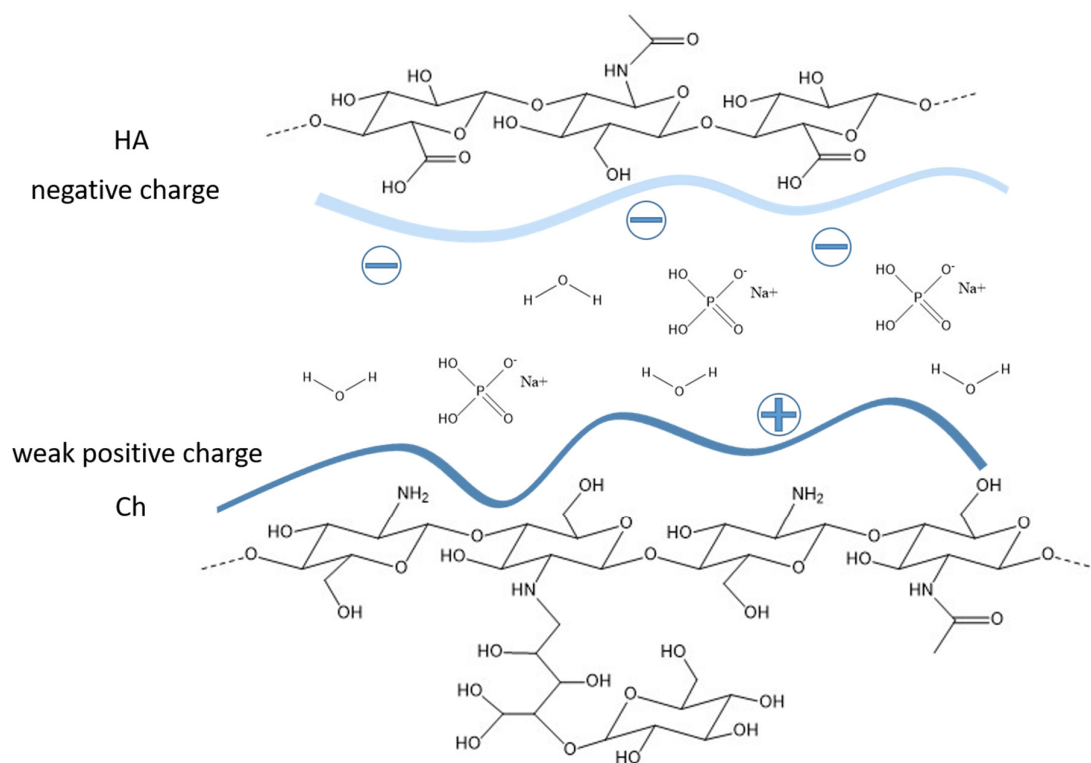

**Figure S1.** Schematic interaction between HA and Ch in Phosphate buffer saline.

Supplement: Supplementary file 1 [file biomolecules-11-00389-s001.pdf]
